# Feasibility Study for Implementing Disease‐Modifying Therapies in Alzheimer's Disease: Process Measures and Patient Pathways across Ireland

**DOI:** 10.1002/alz70858_101630

**Published:** 2025-12-25

**Authors:** Eliza Georgiou, Petya Grigorova, Sevinc Elif Sen, Anne‐Marie Miller, Antoinette O'Connor, Irina Kinchin, Iracema Leroi

**Affiliations:** ^1^ Deparment of Psychiatry, Medical School, University of Patras, Patras, Greece; ^2^ Dementia Trials Ireland, Trinity College Dublin, Dublin, Ireland; ^3^ Dementia Trials Ireland, Dublin, Ireland; ^4^ Centre for Health Policy and Management, Trinity College Dublin, Dublin, Ireland; ^5^ Trinity College Institute of Neuroscience, Trinity College Dublin, Dublin, Ireland; ^6^ Global Brain Health Institute, Dublin, Ireland

## Abstract

**Background:**

Alzheimer's disease (AD) is a leading cause of cognitive decline, and the recent European approval of disease‐modifying therapies (DMTs) like Donanemab and Lecanemab offers new hope for slowing its progression. However, healthcare systems across Europe, including Ireland, are unprepared for their real‐world implementation. Challenges include equitable access, infrastructure readiness, and treatment logistics. This study aims to evaluate the feasibility of implementing DMTs across public and private healthcare sectors in Ireland, positioning it as a stepping stone toward broader European readiness.

**Method:**

This multi‐center observational feasibility study will assess DMT implementation readiness across Irish centers (Dublin, Waterford, Cork, Galway). The study employs patient journey mapping using retrospective and hypothetical data, alongside evaluations of key process measures, including referral timelines, biomarker verification, infusion readiness, and follow‐up protocols. Graph‐theory modeling will visualize patient pathways and highlight system inefficiencies.

**Results:**

The study is expected to reveal disparities in DMT access, logistical bottlenecks, and enablers for scalable implementation. Graph‐based modeling will provide actionable insights to optimize patient pathways and healthcare delivery frameworks. Stakeholder feedback will inform patient‐centered approaches and address equity challenges.

**Conclusion:**

This feasibility study addresses the critical need for preparedness in integrating DMTs for AD, which were recently approved in Europe but remain underutilized due to systemic barriers. By generating actionable insights and laying the groundwork for national guidelines, this project not only advances Ireland's readiness but also serves as a model for other European countries seeking to adopt transformative therapies for Alzheimer's disease.

